# Knowledge mapping of paediatric fever—a visual analysis based on CiteSpace

**DOI:** 10.3389/fped.2024.1383342

**Published:** 2024-05-17

**Authors:** Di Liu, Dingruo Zhang, Tianyuan Yu, Sheng Guo, Xiaona Xue, Hui Hu, Jiayue Liu, Yue Xu, Narentuya

**Affiliations:** ^1^ Department of Acupuncture-Moxibustion, Dongfang Hospital Affiliated to Beijing University of Chinese Medicine, Beijing, China; ^2^ School of Acupuncture-Moxibustion and Tuina, Beijing University of Chinese Medicine, Beijing, China; ^3^ Department of Massage, Dongfang Hospital Affiliated to Beijing University of Chinese Medicine, Beijing, China; ^4^ Department of Paediatrics, Dongfang Hospital Affiliated to Beijing University of Chinese Medicine, Beijing, China

**Keywords:** paediatric fever, CiteSpace, visual analysis, research hotspots, clinical management

## Abstract

**Objective:**

This study aimed to analyse the research hotspots and frontiers in the field of paediatric fever between 2013 and 2023.

**Methods:**

The included articles were visually analysed using CiteSpace 6.1.R6 software.

**Results:**

A total of 2,662 Chinese-language articles and 1,456 English-language articles were included in the study. Based on the Chinese literature, research groups were identified represented by Xinmin Li, Jinling Hong and Hongshuang Luo. Based on the English literature, research groups were formed represented by Henriette Moll, Santiago Mintegi and Elizabeth Alpern. Tianjin University of Traditional Chinese Medicine was the institution with the largest number of publications in the Chinese literature, and the Centers For Disease Control And Prevention was the institution with the largest number of publications in the English literature. The research on paediatric fever mainly focused on mechanism exploration, green treatment and clinical management.

**Conclusion:**

Several relatively stable research groups have been formed. Future studies on the differential diagnosis, rational drug use, standardised management and clinical practice guidelines for paediatric fever are needed.

## 1 Introduction

The severity of paediatric fever varies, with mild fever defined as 37.5°C–38.5°C, moderate fever as 38.5°C–39.5°C and high fever as ≥39.5°C (1, 2). Paediatric fever is a self-protection mechanism triggered by the body to defend against pathogens. Exogenous infections often occur in children due to their underdeveloped immunity. These infections are generally associated with viruses, bacteria and mycoplasma in clinical tests, and the symptoms are exacerbated by many factors, such as congenital diseases and malnutrition. Uneven indoor and outdoor temperatures in the winter and spring seasons or in cold areas are more likely to induce upper respiratory diseases (3). Unlike in adults, the posterior nasal meatus is not completely developed in children, who have relatively narrow lumens, and both local cartilage support in the airway and ciliary movement are weak. With the combined effects of environmental factors, such as air pollution and enclosed spaces, pathogens present in the body are difficult to eliminate, resulting in a protracted course of disease (4). A lack of laboratory indications and standardised medication guidance has led to the widespread use of antibiotics in households, which in the long run may induce drug resistance. The World Health Organization has recommended acetaminophen and ibuprofen as the basic antipyretic drugs in children; however, high doses of these drugs are toxic to the liver and kidneys. In modern medicine, physical cooling is used as an adjunctive therapy, and the illness often relapses. In addition, the media used, such as ice packs and alcohol, may cause local tissue injury (5).

Paediatric fever is currently receiving increasing attention. Related literature is interrelated and complex, and the traditional methods used for summarising and presenting the literature are not intuitive and visible, resulting in a somewhat superficial analysis of the literature. With the rapid development of big data platforms, the ability to summarise and organise the research points of paediatric fever hidden in vast amounts of medical information and apply them to the decision-making process in scientific research has become a hot topic amongst researchers. CiteSpace is a widely used knowledge mapping tool that utilises information technology to calculate and analyse related literature in certain research fields, structure the knowledge development process and visualise the status, hotspots and trends of the research (6, 7).

Large studies on paediatric fever have been published worldwide, in a wide variety of document types (8, 9). However, most of these studies only review knowledge from a certain dimension, and the frontier research has not been sufficiently summarised, making it difficult to find the core contents within the literature quickly. Researchers spend a great deal of time organising and searching for related articles. To understand the research hotspots and trends in the field of paediatric fever quickly and accurately, the present study is designed to scientifically sort and organise the literature using bibliometric methods. The China National Knowledge Infrastructure (CNKI) and the Web of Science (WoS) are chosen as database sources. A knowledge map of research achievements related to paediatric fever is constructed using CiteSpace software, and the research process, current status and development trends in this field are summarised, concluded and predicted. This provides a scientific basis for researchers to grasp future research trends and promote the research and development of this field.

## 2 Materials and methods

### 2.1 Literature retrieval

#### 2.1.1 Search strategy

The Chinese literature included in this study was retrieved from the CNKI database, and the English literature was from the WoS core database. The database retrieval took place between 1 November 2013 and 31 October 2023. The search formulae were set as “(children + juvenile + paediatric + baby + infant + adolescent + minor) AND (pyrexia + fever + antipyretic + antifebrile + elevated body temperature + cooling) AND (exogenous + cold + contagious + infection + virus + bacteria + inflammation)” for the CMKI database and “([TS = (children)] OR [TS = (child)] OR [TS = (paediatric)] OR [TS = (infantile)] OR [TS = (baby)]) AND ([TS = (cold)] OR [TS = (influenza)] OR [TS = (flu)] OR [TS = (exogenous)] OR [TS = (bacteria)] OR [TS = (bacterial infection)]) AND ([TS = (fever)] OR [TS = (pyrexia)] OR [TS = (febris)])” for the WoS database.

#### 2.1.2 Inclusion criteria

(1) The literature was published publicly; (2) the patients were aged ≤18 years; and (3) fever was included as one of the outcome measures.

#### 2.1.3 Exclusion criteria

(1) The literature lacked the fields required for analysis; (2) the literature was a conference paper, systematic review, meta-analysis, review, news article, patent or scientific or technological achievement; or (3) the literature was a repeated publication.

During the process of literature screening, the data were entered by two people. In the case of uncertainty, the data were determined by a third person and then corrected.

### 2.2 Data export

All literature records and citations retrieved from the WoS database were exported based on the preset keywords in plain text format, and those retrieved from the CNKI database were exported based on the preset keywords in RefWorks format. CiteSpace provides a visual narrative that uncovers the research frontiers, research hotspots, cluster analysis and potential future directions (10, 11). In this study, a knowledge map of research achievements related to paediatric fever was constructed using CiteSpace and VOSViewer software.

### 2.3 Statistical analysis

The text document was named “download_n.txt”, and data of the literature, including authors, countries, institutions, keywords and co-citations, were converted and analysed using CiteSpace 6.2.R4, with the following parameters: Timespan = 2013–2023, SliceLength = 1 and TopN = Top 50. The pruning strategies included pathfinder, pruning networks and pruning the merged network, and the remaining parameters were set to default.

## 3 Results

### 3.1 Annual publications

**Figure 1:**
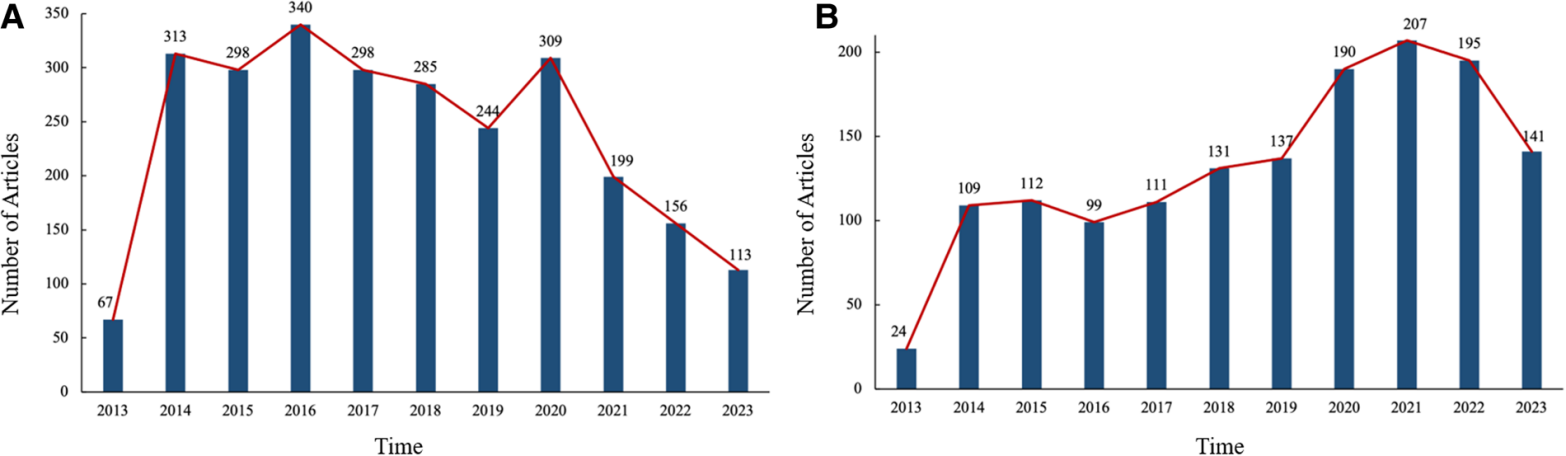
Annual publications of literature on the research of pediatric fever. (**A**) number of annual publications of Chinese literature on the research of pediatric fever; and (**B**) number of annual publications of English literature on the research of pediatric fever.

A total of 2,662 articles related to paediatric fever were retrieved from the CNKI database. A quantitative analysis of annual publications showed that the number of publications was relatively low in 2013 and increased rapidly from 2014, with more than 240 publications every year. The number of annual publications peaked in 2016, with a total of 340 articles published, and has decreased every year since 2021 (Figure 1A).

A total of 1,456 articles related to paediatric fever were retrieved from the WoS database. A quantitative analysis of annual publications showed that the number of publications was relatively low in 2013 and increased rapidly from 2014, with approximately 100 articles published every year. The number of annual publications peaked in 2021, with a total of 207 articles published, and decreased each year in 2022 and 2023 (Figure 1B).

### 3.2 Country/regional distribution of publications

**Figure 2:**
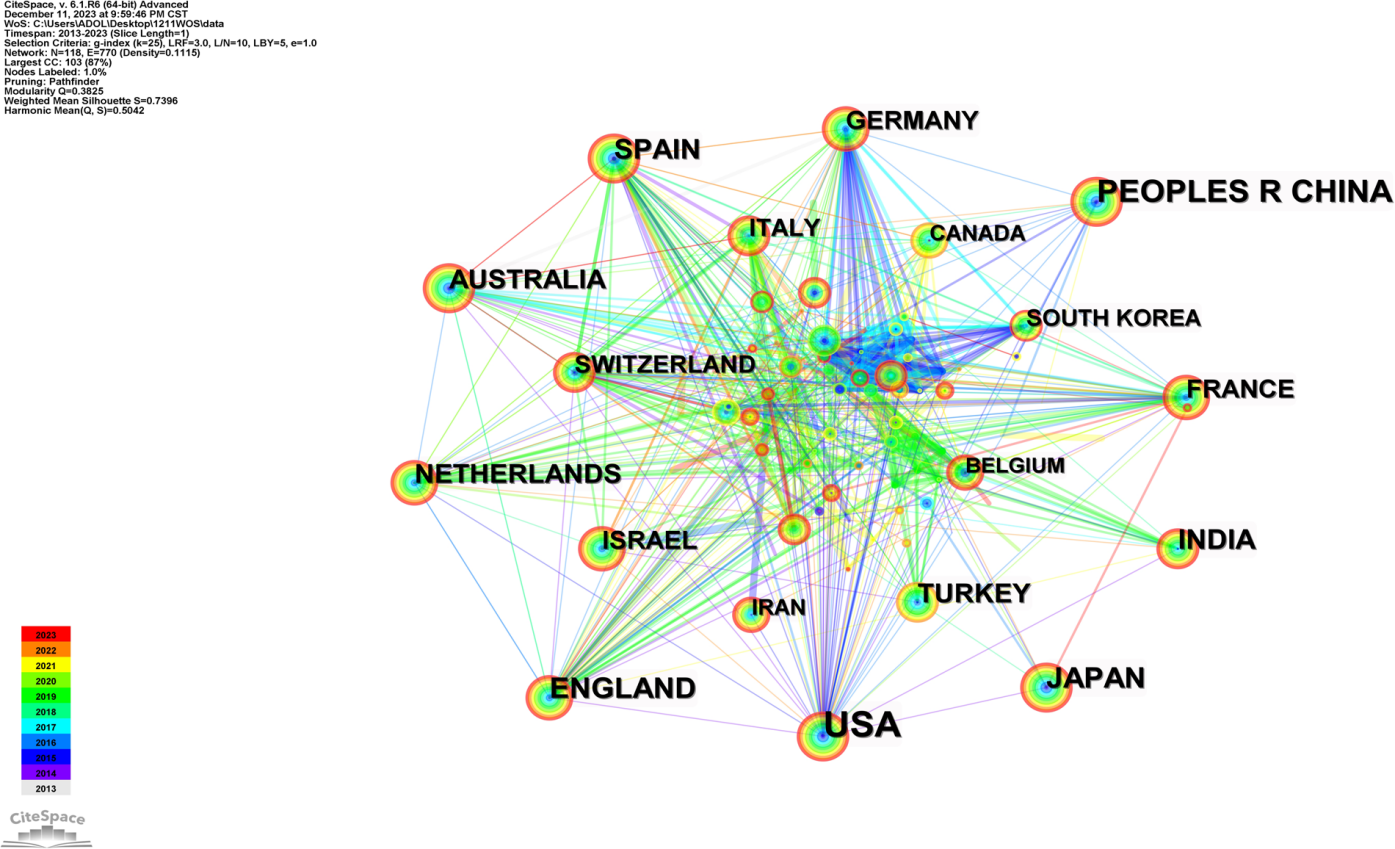
Country/regional distribution of publications on the research of pediatric fever.

Statistical analysis of the country/regional information on the research of paediatric fever in the WoS database showed that between 2013 and 2023, the United States published the largest number of articles (351 articles), followed by China and Japan, with 196 and 103 articles, respectively. In addition, the United Kingdom and India each published more than 100 articles (Figure 2).

### 3.3 Authors

Authors of Chinese publications were analysed, where a node represents an author of the article and the label shows the name of the author. The node size and the font size of the label represent the number of related publications by the author. A larger label indicates more publications. The line between nodes represents the cooperation between authors, and the thickness of the line represents the strength of the connection, with a thicker line indicating stronger cooperation. The colour of the lines corresponds to the year information shown in the colour chart on the bottom left of the figure, indicating the year of the first cooperation between the authors. The nodes and labels are generally dense in the figure, indicating a large number of authors involved in research related to paediatric fever.

**Figure 3:**
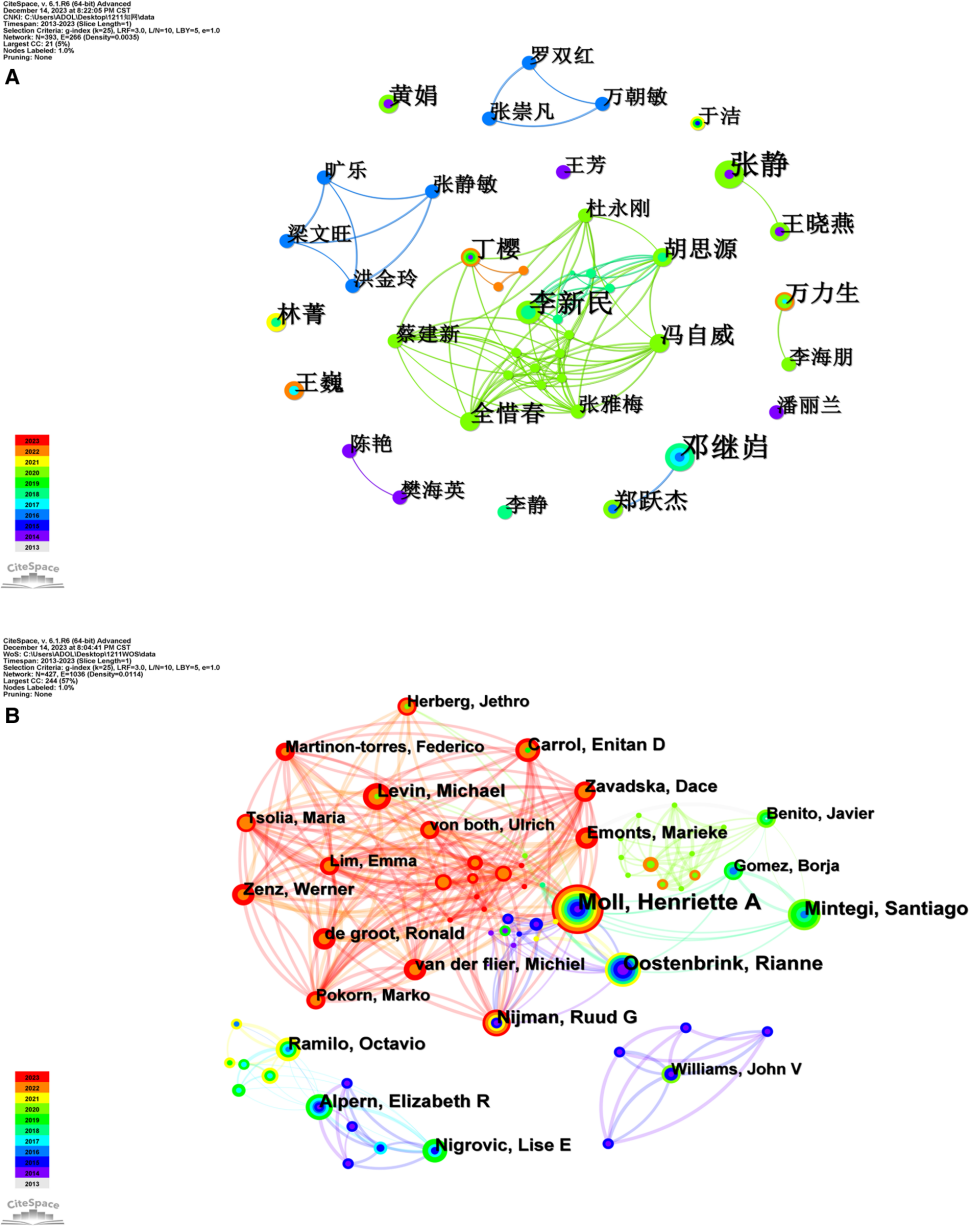
Cooperation network of authors of publications related to the research of pediatric fever. (**A**) cooperation network of authors of Chinese literature; and (**B**) cooperation network of authors of English literature on the research of pediatric fever.

As shown in Figure 3A, 393 nodes and 266 lines were obtained, with a correlation density of 0.0035. The authors with relatively more cooperation included Xinmin Li, Siyuan Hu, Ziwei Feng, Xichun Quan, Jianxin Cai and Yamei Zhang in the centre of the figure, and they form a cooperative network. In addition, there were two other cooperative networks formed in 2016. One network, which was first formed in 2016, consisted of Jinling Hong, Jingmin Zhang, Wen Wang Liang and Le Kuang, and the other network consisted of Shuanghong Luo, Chaomin Wan and Chongfan Zhang. However, there were few or even no connections among these three cooperative networks or between these three cooperative networks and other authors, indicating the coexistence of both independent and cooperative studies.

As shown in Figure 3B, 427 nodes and 1,036 lines were obtained, with a correlation density of 0.0114. Authors with a high centrality included Henriette Moll, Santiago Mintegi and Elizabeth Alpern, and cooperation was noted among many authors. The core authors among others included Henriette Moll, who had published 19 articles. This author has been focusing on paediatric fever since 2014 and closely cooperated with others in the research of paediatric fever in 2023, as shown in the upper left of the figure.

### 3.4 Institutions

The institutions in the Chinese publications were analysed; nodes represent the institutions and lines represent the collaboration between institutions. The colour chart corresponds to the year of the collaboration between institutions, and the size of the node reflects the relative number of articles published by the institution, with a thicker line indicating a closer collaboration between institutions.

**Figure 4:**
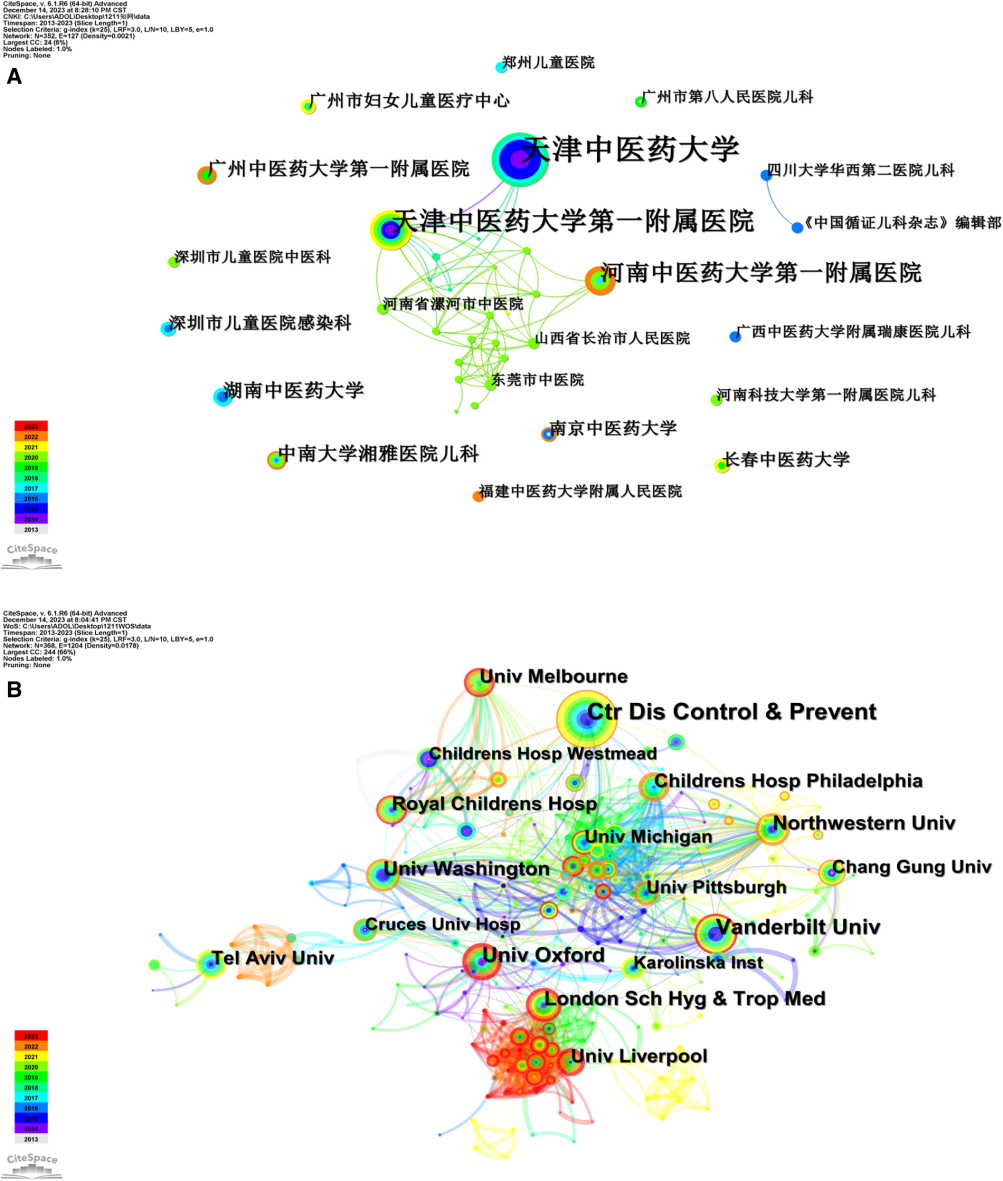
Collaboration network of institutions of publications related to the research of pediatric fever. (**A**) collaboration network of institutions of Chinese literature on the research of pediatric fever; and (**B**) collaboration network of institutions of English literature on the research of pediatric fever.

The top 21 institutions with the most publications in Chinese literature between 2013 and 2023, including co-ranked institutions, were selected. The top three institutions in China in terms of the total amount of publications on paediatric fever included Tianjin University of Chinese Medicine, the First Affiliated Hospital of Tianjin University of Chinese Medicine and the First Affiliated Hospital of Henan University of Chinese Medicine. These three institutions closely collaborated with the Luohe Hospital of Traditional Chinese Medicine in Henan Province, Changzhi People's Hospital in Shanxi Province and Dongguan TCM Hospital between 2018 and 2020. The node on the upper right of Figure 4A reflects the close collaboration between the paediatrics department of West China Second University Hospital of Sichuan University and the editorial department of the Chinese Journal of Evidence-Based Paediatrics in 2016. In the remaining institutions, more independent research is observed, with less collaboration with other institutions and the top three institutions.

Institutions that published literature in English were analysed, and it was found that the institutions that published the most articles on paediatric fever included the Centers For Disease Control And Prevention, Vanderbilt University, Oxford University and the London School of Hygiene and Tropical Medicine, and the institutions with the highest centrality was the London School of Hygiene and Tropical Medicine (0.17). As shown at the bottom left of Figure 4B, this institution closely collaborated with other institutions, such as Liverpool University in 2023.

### 3.5 Keywords

#### 3.5.1 Keyword co-occurrence analysis

Keywords reflect and guide the main content and show for the first time the interesting points of the article. In the keyword co-occurrence map, each node represents a keyword captured. A larger node diameter indicates a higher frequency of the keyword. A line connecting two nodes indicates a co-occurrence relationship between the two keywords, and a thicker line suggests a stronger co-occurrence.

**Figure 5:**
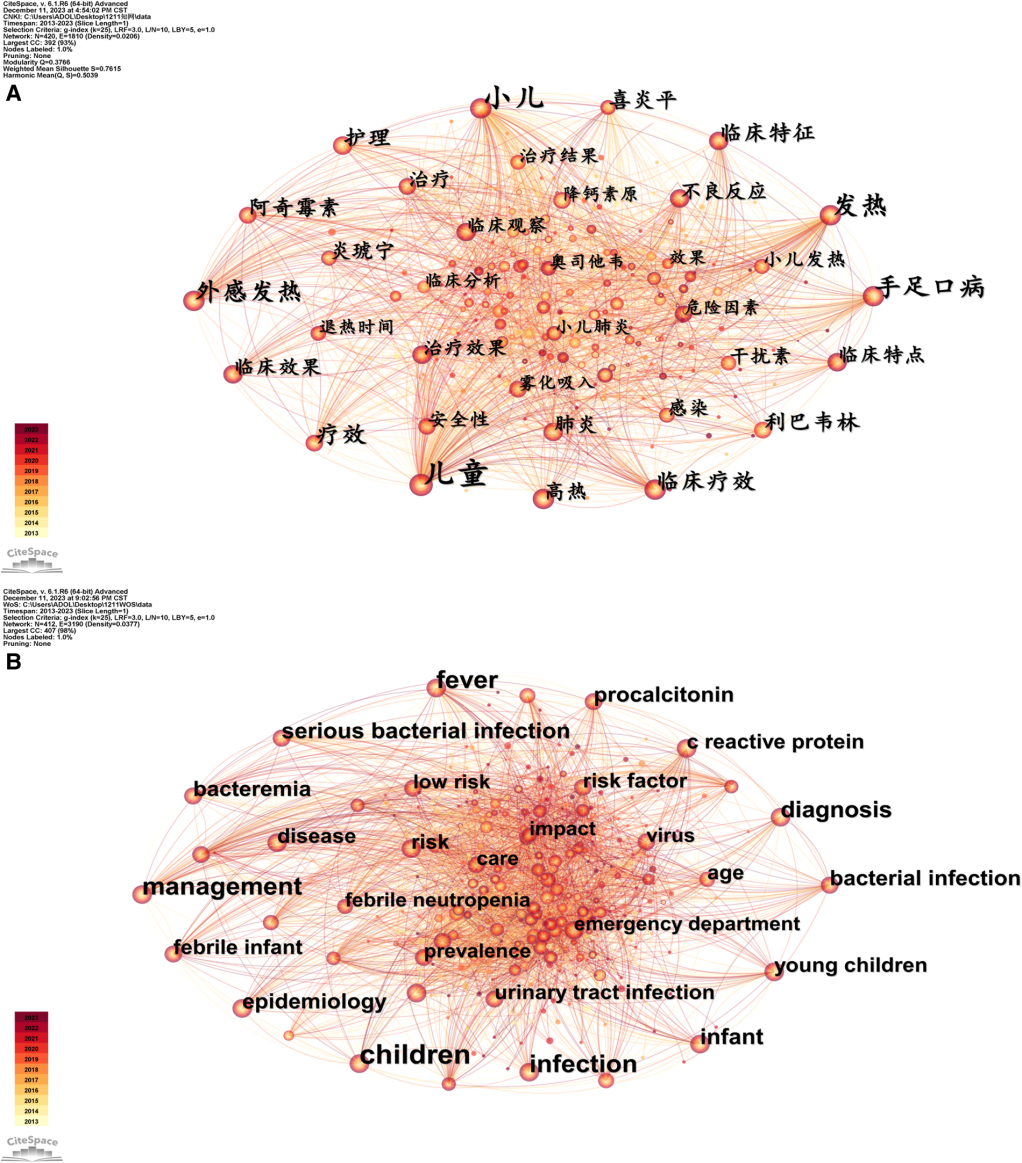
Keyword co-occurrence in literature on the research of pediatric fever. (**A**) keyword co-occurrence network of Chinese literature on the research of pediatric fever; and (**B**) keyword co-occurrence network of English literature on the research of pediatric fever.

Among the keywords in the Chinese and English literature, the words with the highest frequencies included “children” and “fever”. In addition to the keywords used as literature screening criteria, 420 nodes were captured in the keyword co-occurrence map for Chinese literature, and those closely related to this study with a higher frequency included “hand-foot-and-mouth disease” (170 times) and “efficacy” (128 times). In the English literature, there were 412 nodes captured in the keyword co-occurrence map, and those closely related to the present study with a higher frequency included “infection” (209 times) and “management” (205 times) (Figure 5).

#### 3.5.2 Keyword clustering analysis

The module value (*Q*-value) and average contour value (*S*-value) of keyword clustering were used to evaluate the significance of mapping. A *Q*-value of >0.3 suggests the clustering structure is significant, an *S*-value of >0.5 is considered reasonable clustering and >0.7 is considered convincing clustering. A smaller cluster number indicates a larger scale.

**Figure 6:**
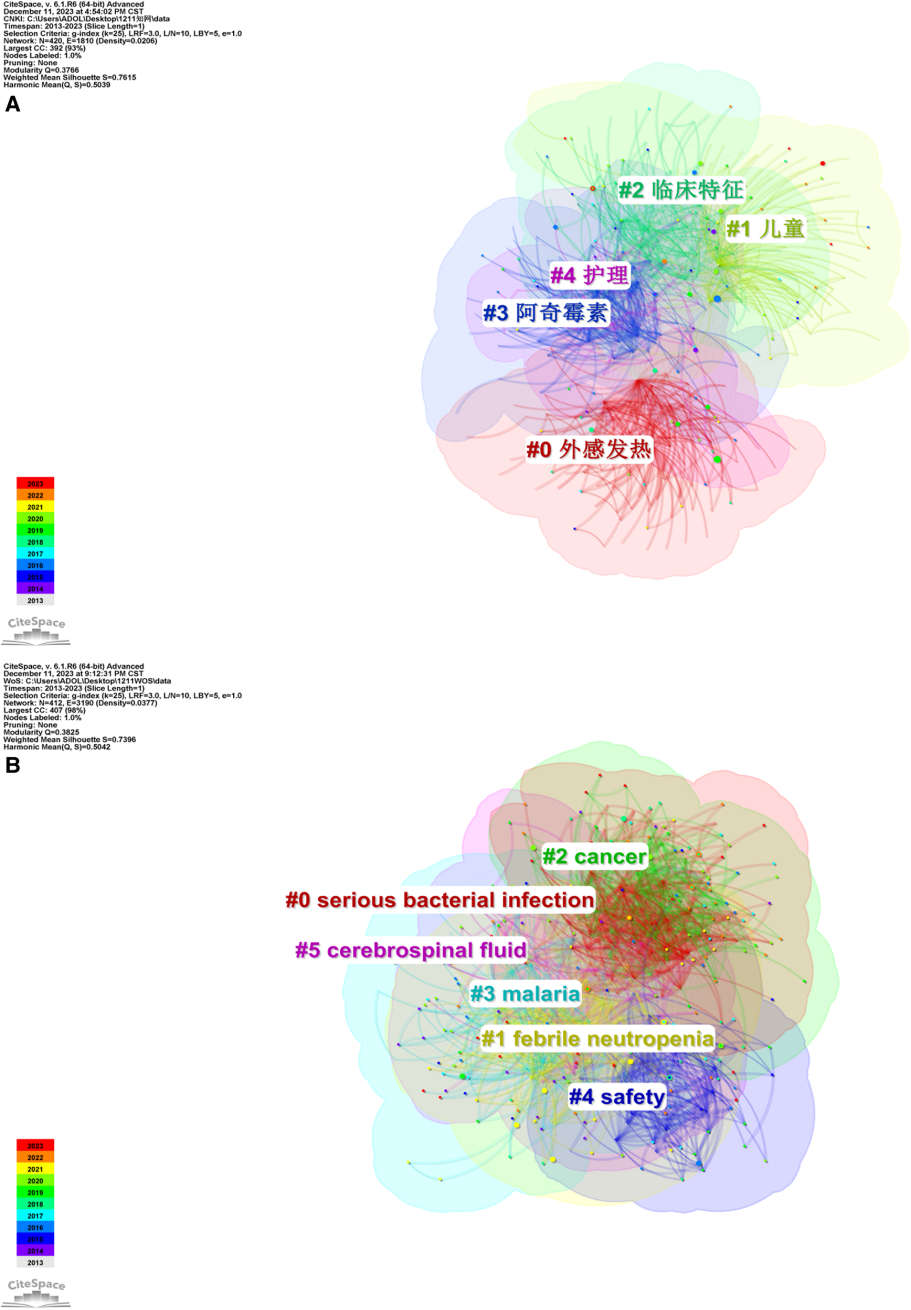
Keyword cluster in Chinese literature on the research of pediatric fever. (**A**) keyword cluster in Chinese literature on the research of pediatric fever; and (**B**) keyword cluster in English literature on the research of pediatric fever.

A total of four modules were obtained in Chinese literature by keyword clustering analysis (Table 1). The analysis showed that *Q* = 0.3766, which was >0.3, indicating that the cluster module structure was limited and the result was reasonable, and *S* = 0.7615, which was >0.7, indicating that the clustering was homogeneous and the result was convincing. The clustering results involved four main research directions, including clusters #0 exogenous fever, #1 children, #2 clinical features, #3 azithromycin and #4 nursing. In the Chinese literature, cluster #0 external fever contained the keyword “infantile massage”, indicating that infantile massage was an important topic in the field of paediatric fever, and this cluster mainly focused on the clinical efficacy and mechanism studies on paediatric fever; cluster #1 focused on the detection of biomarkers for paediatric fever; cluster #2 included epidemiological studies, covering fever-related diseases such as hand-foot-and-mouth disease and pneumonia; cluster #3 focused on symptomatic treatment with drugs, among which azithromycin, Xiyanping, ribavirin and potassium sodium dehydroandrographolide succinate were highly captured in the literature statistics; the core keywords in cluster #4 were “nursing” and “emergency treatment”, including infant fever care and febrile convulsion (Figure 6A).

**Table 1:** Information on Chinese keyword clustering for research of pediatric fever.

Cluster number	Cluster size	Contour value	Year	Clustering labels(LLR)
0	104	0.781	2016	Exogenous fever (258.94, 1.0 × 10^−4^); pediatric (120.75, 1.0 × 10^−4^); children (71.6, 1.0 × 10^−4^); infantile massage (49.92, 1.0 × 10^−4^); clinical observation (43.75, 1.0 × 10^−4^)
1	86	0.605	2017	Children (185.56, 1.0 × 10^−4^); fever (125.6, 1.0 × 10^−4^); pediatric (59.3, 1.0 × 10^−4^); procalcitonin (58.5, 1.0 × 10^−4^); C-reactive protein (35.01, 1.0 × 10^−4^)
2	82	0.787	2016	clinical features (112.79, 1.0 × 10^−4^); hand-foot-and-mouth disease (54.98, 1.0 × 10^−4^); pneumonia (54.66, 1.0 × 10^−4^); epidemiology (44.87, 1.0 × 10^−4^); treatment (42.68, 1.0 × 10^−4^)
3	78	0.867	2016	azithromycin (79.64, 1.0 × 10^−4^); xiyanping (77.12, 1.0 × 10^−4^); efficacy (76.92, 1.0 × 10^−4^); ribavirin (61.13, 1.0 × 10^−4^); potassium sodium dehydroandrographolide succinate (61, 1.0 × 10^−4^)
4	42	0.786	2015	nursing (77.79, 1.0 × 10^−4^); hyperpyrexia (74.9, 1.0 × 10^−4^); emergency treatment(33.26, 1.0 × 10^−4^); clinical nursing(33.26, 1.0 × 10^−4^); febrile seizure (28.49, 1.0 × 10^−4^)

A total of five modules were obtained in the English literature by keyword clustering analysis (Table 2). The analysis showed that *Q* = 0.3825, which was >0.3, indicating that the cluster module structure was limited and the result was reasonable, and *S* = 0.7396, which was >0.7, indicating that the clustering was homogeneous and the result was convincing. The clustering results in the English literature included the following clusters: #0 serious bacterial, #1 febrile neutropenia, #2 cancer, #3 malaria, #4 safety and #5 cerebrospinal fluid. Cluster #0 serious bacterial infections included emergency treatment of children with fever and procalcitonin tests; cluster #1 described epidemiological studies involving febrile neutropenia, respiratory syncytial virus infection, influenza virus and other thermogenic factors; cluster #2 included various types of paediatric fever, mainly cancer-related fever; cluster #3 involved malaria fever and extended studies on antimicrobial resistance under this category, in which gastroenteritis diseases, such as diarrhoea, were presented in addition to fever; cluster #4 focused on the safety factors of febrile diseases in paediatric fever, and vaccine safety was also widely concerned in addition to infection factors; in cluster #5, case reports emphasised the importance of cerebrospinal fluid diagnosis in critically ill fever in children and focused on the disease types and detection methods associated with particular cases (Figure 6B).

**Table 2:** Information on English keyword clustering for research of pediatric fever.

Cluster number	Cluster size	Contour value	Year	Clustering labels(LLR)
0	88	0.76	2016	Serious bacterial infection (85.84, 1.0 × 10^−4^); fever (50.67, 1.0 × 10^−4^); emergency department (47.48, 1.0 × 10^−4^); procalcitonin (45.58, 1.0 × 10^−4^); febrile infant (38.45, 1.0 × 10^−4^)
1	83	0.706	2016	Febrile neutropenia (27.89, 1.0 × 10^−4^); respiratory syncytial virus (23.44, 1.0 × 10^−4^); human metapneumovirus (23.39, 1.0 × 10^−4^); respiratory viruses (22.94, 1.0 × 10^−4^); epidemiology (22.57, 1.0 × 10^−4^)
2	67	0.757	2017	Febrile neutropenia (102.38, 1.0 × 10^−4^); cancer (26.62, 1.0 × 10^−4^); influenza (25.14, 1.0 × 10^−4^); neutropenia (20.37, 1.0 × 10^−4^); bloodstream infection (15.87, 1.0 × 10^−4^)
3	67	0.706	2017	malaria (37.42, 1.0 × 10^−4^); africa (27.57, 1.0 × 10^−4^); gastroenteritis (19.68, 1.0 × 10^−4^); antimicrobial resistance (19.53, 1.0 × 10^−4^); diarrhea (19.53, 1.0 × 10^−4^)
4	58	0.788	2016	Safety (46.82, 1.0 × 10^−4^); influenza vaccine (42.9, 1.0 × 10^−4^); immunogenicity (27.25, 1.0 × 10^−4^); seasonal influenza (23.35, 1.0 × 10^−4^); serious bacterial infection (19.6, 1.0 × 10^−4^)
5	44	0.723	2017	Cerebrospinal fluid (25.86, 1.0 × 10^−4^); case report (20.67, 1.0 × 10^−4^); sickle cell disease (15.49, 1.0 × 10^−4^); osteomyelitis (15.49, 1.0 × 10^−4^); cytokines (11.15, 0.001)

#### 3.5.3 Keyword burst analysis

A keyword burst refers to a sudden change in the frequency of a certain keyword during a particular time period, representing a new trend or turning point in related fields or directions. This can be used as a reference for future research directions.

**Figure 7:**
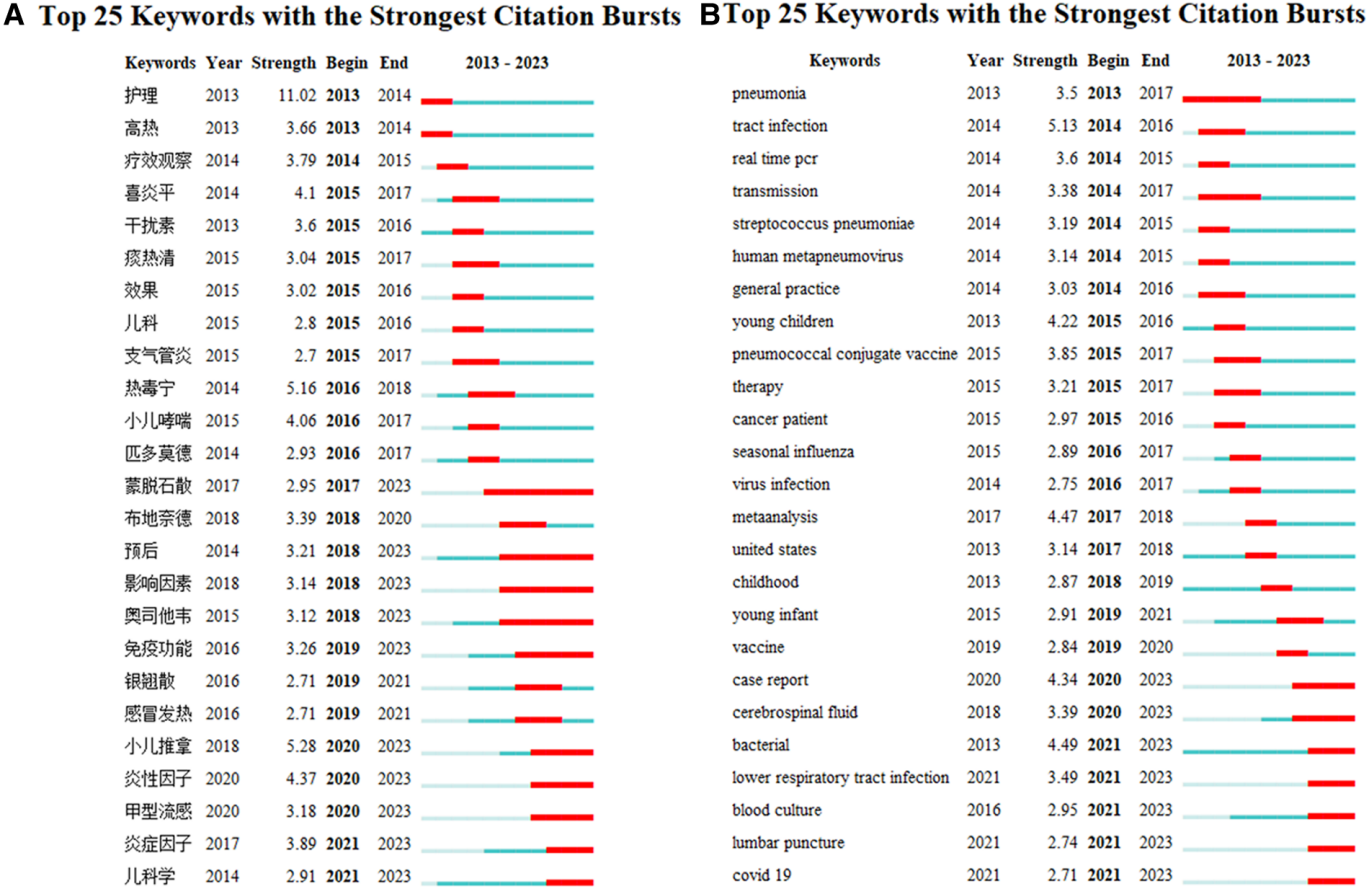
Keywords burst in literature on the research of pediatric fever. (**A**) keyword burst map for Chinese literature on the research of pediatric fever; and (**B**) keyword burst map for English literature on the research of pediatric fever.

The top 25 keywords with the strongest citation bursts in the Chinese literature on paediatric fever research between 2013 and 2023 are shown in Figure 7A and sorted according to the time of the earliest occurrence. The columns on the left side include keywords, years, strength, start year and end year, and the horizontal line on the right side represents the period of all studies (i.e., between 2013 and 2023). Blue–green denotes the absence of a keyword burst, and red denotes the presence of a keyword burst. It can be seen from the map that the research hotspots in the past three years include infantile massage, influenza A, inflammatory factors and paediatrics.

As shown in Figure 7B, the top three keywords with the strongest citation burst in English literature were “tract infection”, “bacterial” and “meta-analysis” with strengths of 5.13, 4.49 and 4.47, respectively. The start and end years of the top three keywords were 2014–2016, 2021–2023 and 2017–2018, respectively. “Bacterial” remained a research hotspot until 2023. The longest duration of a burst was found in the keyword “pneumonia”, where the start and end years of this keyword burst were 2013–2017. The latest keywords with citation bursts were “bacterial”, “lower respiratory tract infection”, “blood culture”, “lumbar puncture” and “COVID-19”, which started in 2021 and continued to 2023. This group of keywords is likely to continue in future research.

#### 3.5.4 Keyword timeline analysis

Changes in keywords in different clusters can be visualised by a keyword timeline map. A timeline map of keyword clustering was constructed with the year of article publication as the x-axis, the keyword cluster number as the y-axis and a slice length of 1 year.

**Figure 8:**
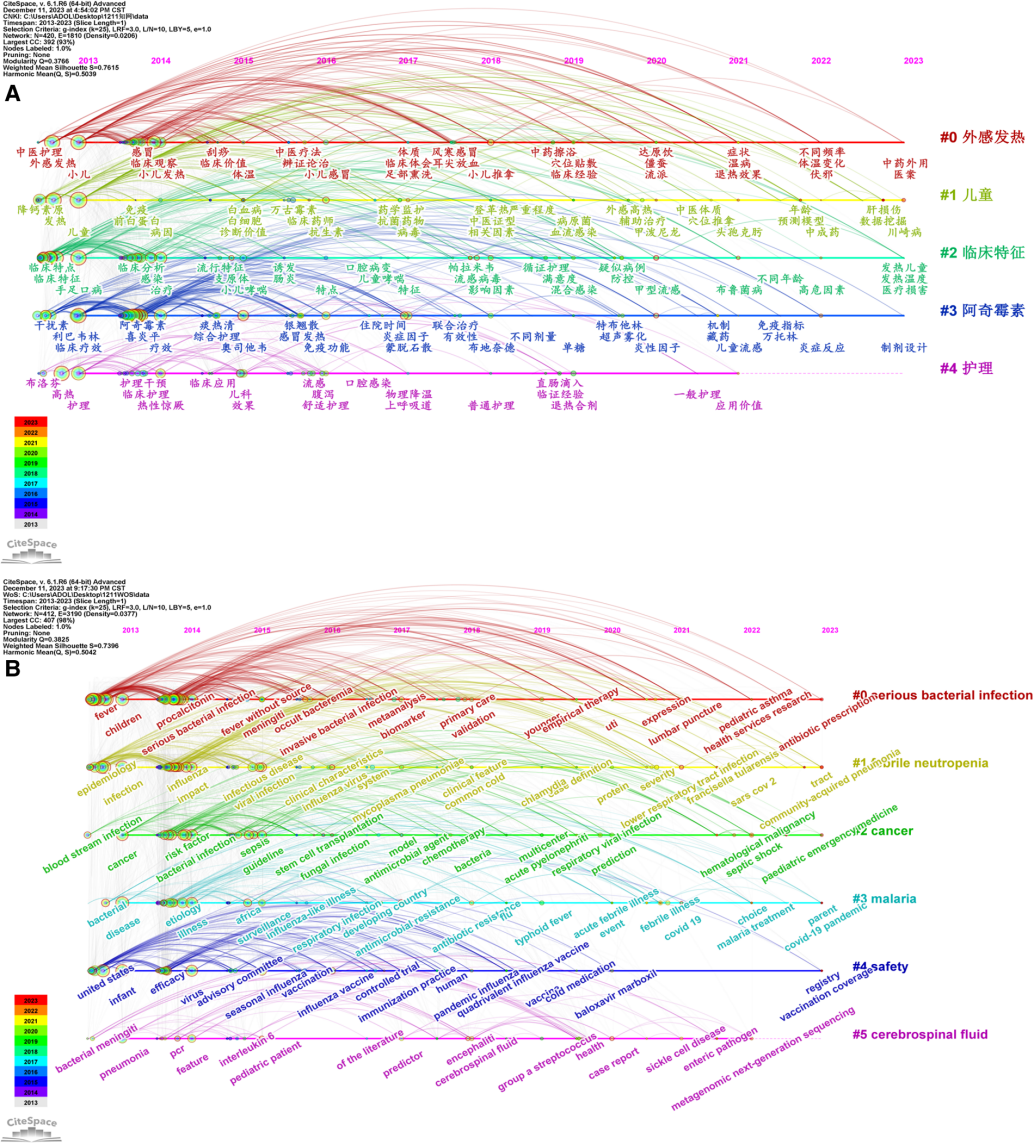
Timeline of literature on the research of pediatric fever. (**A**) timeline chart of Chinese literature on the research of pediatric fever; and (**B**) timeline chart of English literature on the research of pediatric fever.

In the Chinese literature, as shown in Figure 8A, in cluster #0, research on exogenous fever focused on clinical observation in 2014; the first clinical study on physique was carried out in 2017, and efforts were also made between 2017 and 2023 on studies of external treatment methods, such as foot fumigation, ear tip bleeding and acupoint application; in cluster #1, clustering analysis under the keyword “children” initially focused on procalcitonin in 2013, and studies on vancomycin increased between 2014 and 2015; in cluster #2, studies on clinical features were featured since 2013; in cluster #3, studies results on the utilisation of azithromycin had accumulated since 2014, and the core period was between 2014 and 2017, during which the hotspots also included the traditional Chinese medicines “Tanreqing” and “Yinqiao powder” and the western medicines “Xiyanping” and “oseltamivir”; in cluster #4, research on nursing started in 2012 and continued to 2021, and the research enthusiasm on this topic remained relatively high between 2013 and 2016 and then declined.

In the English literature, as shown in Figure 8B, in cluster #0 serious bacterial, in-depth studies on procalcitonin were conducted in 2014, meta-analyses of biomarkers were conducted in 2017 and lumbar puncture was introduced as a hotspot in 2021; in cluster #1 febrile neutropenia, this disease was associated with influenza, and mycoplasma and chlamydia pneumonia were studied in 2014, and it was associated with SARS-CoV-2 in 2022; in cluster #2 cancer, risk factors were explored, followed by studies on stem cell transplantation and antimicrobials, and later studies were extended to septic shock in haematologic malignancies; in clustering #3 malaria, aetiology studies, typhoid fever and febrile diseases were initially emphasised, and then treatment became the hotspot; in cluster #4 safety, pandemic influenza was concerned in 2018 and baloxavir malbosartate was studied in 2020; and in cluster #5 cerebrospinal fluid, interleukin-6 was mainly studied in 2015, and Group A streptococcus was explored in 2019–2020, followed by the studies on the correlation of intestinal pathogens with sickle cell disease.

#### 3.5.5 Keyword time zone analysis

**Figure 9:**
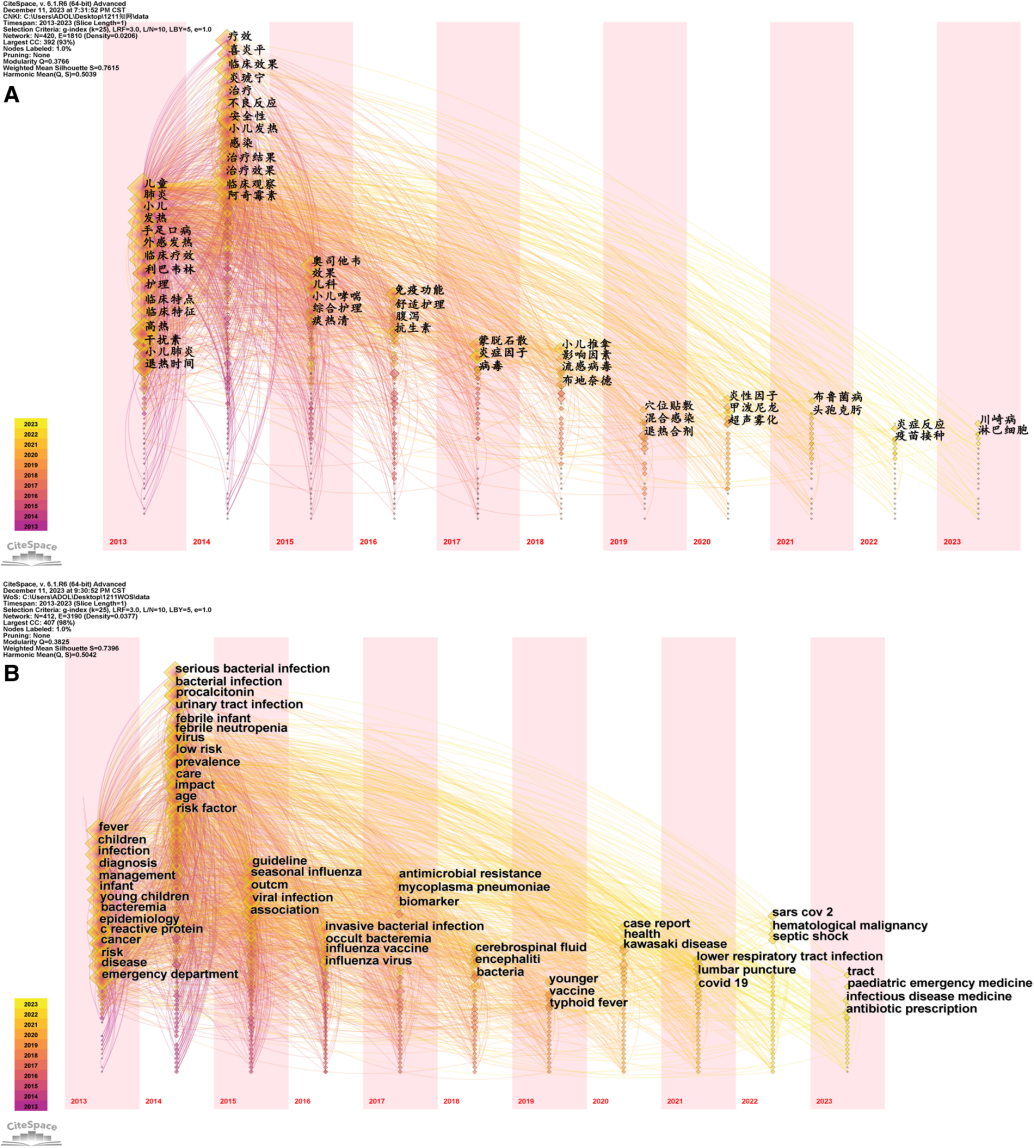
Time zone of literature on the research of pediatric fever. (**A**) time zone chart of Chinese literature on the research of pediatric fever; and (**B**) time zone chart of English literature on the research of pediatric fever.

A keyword co-occurrence time zone map was constructed by using CiteSpace to organise and explore the development process of paediatric fever. The literature between 2013 and 2023 was retrieved. The map was drawn based on the year in which the keyword first appeared to show the changes and development process of the research of paediatric fever from the time dimension. Studies related to paediatric fever, from the initial clinical symptoms of the disease to the exploration of the immune mechanism, and from the treatment with oral antibiotics to the utilisation of external treatment options, such as massage and acupoint application, are summarised in Figure 9A. They reflect the research progress from macroscopic to microscopic studies, the expansion of treatment methods and the development from clinical studies to both mechanism and clinical studies.

As shown in Figure 9B, studies related to paediatric fever emphasised the diagnosis of the disease and bacteria in 2013; various studies were conducted, with infection as a research hotspot in 2014; studies focused on influenza in 2015; research on vaccines started in 2016; studies were expanded to brain infection in 2018; extensive studies on febrile diseases in children, including Kawasaki disease and lower respiratory tract infections, have been conducted since 2019, and efforts were also made on the prescription of paediatric emergency drugs and antibiotics.

### 3.6 Co-citation analysis

**Figure 10:**
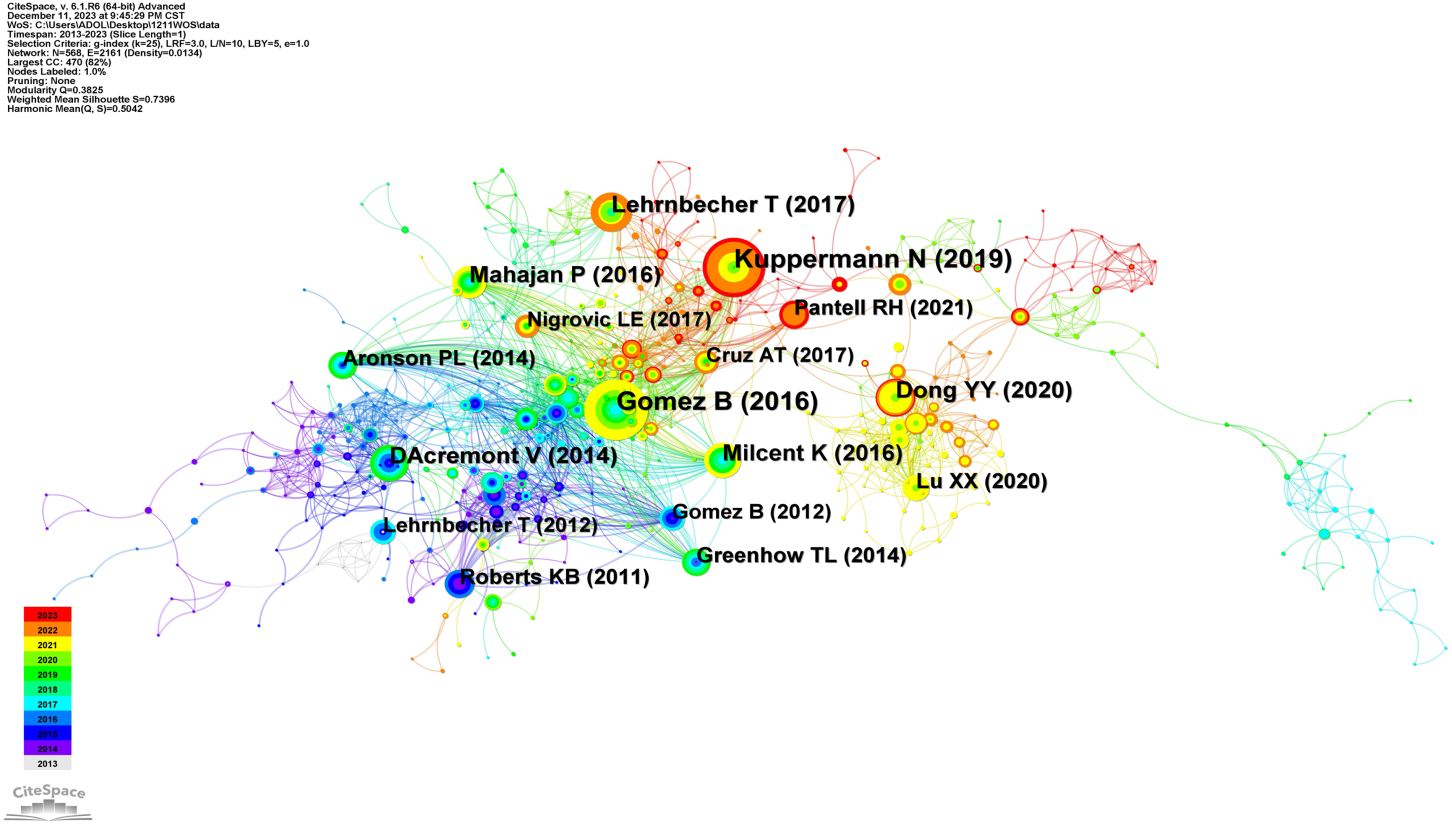
Co-citation chart of English literature on the research of pediatric fever.

Author co-citations referring to two or more authors were cited by one or more subsequent articles at the same time, and these two or more authors constituted a co-citation relationship. In Figure 10, the nodes denote the citation frequencies, and the size of the nodes is proportional to the citation frequency. The top three authors with the highest citation frequency were Borja Gomez, Nathan Kuppermann and Thomas Lehrnbecher, with centralities of 0.07, 0.15 and 0.16, respectively. The citation frequency of the third author was not the highest; however, the centrality of this author (0.16) was ranked first, suggesting that his publications were of academic significance and can be used as key references (Table 3).

**Table 3:** High-frequency co-cited English literature on the research of pediatric fever.

Number	Frequency	Authors	Journals	References
1	45	Gomez B.	PEDIATRICS	Validation of the “Step-by-Step” Approach in the Management of Young Febrile Infants
2	44	Kuppermann N.	JAMA PEDIATR	A Clinical Prediction Rule to Identify Febrile Infants 60 Days and Younger at Low Risk for Serious Bacterial Infections
3	29	Lehrnbecher T.	J CLIN ONCOL	Guideline for the Management of Fever and Neutropenia in Children With Cancer and Hematopoietic Stem-Cell Transplantation Recipients: 2017 Update

### 3.7 Citation analysis

**Figure 11:**
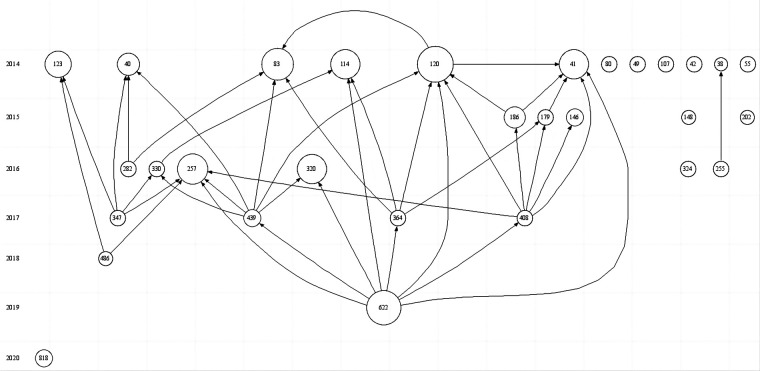
Generate a citation chronological chart through visual analysis.

Generate a citation chronological chart through visual analysis (Figure 11). Screen out the top 30 most important literature in this field and rank them by LCS (local citation score). The larger the circle, the more times it is cited, and the arrow indicates the citation relationship between references. The number indicates the article's numbering in the citation library. As shown in Table 4, the three most cited articles are Aronson PL's study published in 2014, Kuppermann N's study published in 2019, and Greenhow TL's study published in 2014. These 3 studies focus on children born under 90 days with fever.

**Table 4:** The citation frequency and citation relationship between references.

Number	Citation library number	Author	Journal	Research	LCS
1	120	Aronson PL	PEDIATRICS	Variation in Care of the Febrile Young Infant <90 Days in US Pediatric Emergency	47
2	622	Kuppermann N	JAMA PEDIATRICS	A Clinical Prediction Rule to Identify Febrile Infants 60 Days and Younger at Low Risk for Serious Bacterial Infections	44
3	83	Greenhow TL	PEDIATRIC INFECTIOUS DISEASE JOURNAL.	The Changing Epidemiology of Serious Bacterial Infections in Young Infants	37

## 4 Discussion

In the present study, literature in the field of paediatric fever was retrospectively analysed. The results were visualised using CiteSpace software to provide a whole picture in multiple dimensions of the research status in the field of paediatric fever as well as the research hotspots and trends.

The period covered in the study was 2013–2023. In 2013, few related articles in the Chinese and English languages were published and the field of paediatric fever was in the exploratory stage. The number of related publications increased significantly in 2014 and increased every year thereafter, indicating that the field of paediatric fever has gradually been featured and become a research hotspot. During the period between 2021 and 2023, the number of publications relatively decreased. Considering the social phenomenon at that time, the possible reason behind this was that more researchers focused their interests on susceptible diseases related to coronavirus because of the COVID-19 pandemic. In Chinese literature, Jing Zhang and Jikui Deng were the authors with the most articles published (six each), and their studies on paediatric fever were relatively far-reaching. The institutions with a large number of publications included two academic institutions of traditional Chinese medicine (Tianjin University of Traditional Chinese Medicine and the First Affiliated Hospital of Tianjin University of Traditional Chinese Medicine). Therefore, paediatric fever appears to have become a hot topic of concern in colleges and universities of traditional Chinese medicine, and related studies were supported by Tianjin University of Traditional Chinese Medicine. In English literature, Henriette Moll had the highest number of publications, with a total of 19 articles published. There were six authors who each published >10 articles. The institutions with a high volume of publications included the Centers for Disease Control and Prevention in the United States (36 articles), which is an authoritative research institution for paediatric fever, followed by Vanderbilt University (25 articles), which is a private university in the United States with paediatrics and premedicine as its dominant specialities.

Author cooperation network analysis revealed three relatively independent research groups in the Chinese literature. Group 1 was composed of Xinmin Li, Siyuan Hu, Ziwei Feng, Yamei Zhang, Xichun Quan, Jianxin Cai, Ying Ding and Yonggang Du. The research interest of this group included the combined treatment of traditional Chinese medicine and Western medicine for inflammatory diseases related to the respiratory system in children; Group 2 was composed of Wen Wang Liang, Le Kuang, Jingmin Zhang and Jinling Hong, and this group conducted insightful studies on drug treatment for febrile seizures in children; Group 3 included Chongfan Zhang, Shuanghong Luo and Chaomin Wan. This group was interested in the treatment of critical illness and the management of bacterial inflammation in children. In addition, some independent authors have conducted in-depth studies in the field of paediatric fever. In the English literature, it was found that several closely connected research groups have made significant achievements in the field of paediatric fever. These groups included those led by Michael Levin, Werner Zenz, Dace Zavadska, Marieke Emonts and Emma Lim. Their studies, focusing on paediatric multisystem inflammatory syndrome caused by known or unknown pathogens, were published mainly in 2022 and 2023. The author with the most publications was Henriette Moll, and she participated in many studies on paediatric infectious diseases. The results of the author cooperation network analysis revealed group-based research in the field of paediatric fever. However, the connections among research groups were not close, with only relatively scattered cooperation among authors. In addition, new progress in the research of paediatric fever was rarely reported in China in recent years. On this basis, close cooperation within the individual group and among different groups is recommended in the field of paediatric fever. Different research groups in the same field have their unique research directions, and their cooperation helps to strengthen interdisciplinary communication and enhance the development of scientific research ideas.

Keywords concisely and accurately summarise the main content of an article. The research hotspots, trends and directions in the field can be identified by sorting and mapping the keywords. The keywords of relevance in both the Chinese and English literature included “fever detection methods”, “epidemiological investigation” and “emergency measures”. Epidemiological studies provide important information for disease control and prevention by analysing the distribution, incidence and transmission patterns of diseases. After understanding the transmission routes and risk factors of diseases, corresponding prevention strategies can be developed, which help reduce the incidence and transmission risk of diseases. Epidemiological studies help to reveal the causes and risk factors of disease. Possible factors contributing to the development of diseases can be identified by analysing the distribution of diseases in a population, which provides insights into their biological and environmental mechanisms. An epidemiological working group was established by the American Academy of Pediatrics under the commission of the Agency for Healthcare Research and Quality to develop practice guidelines for the evaluation and management of children aged 8–60 days with fever, and 21 action guidelines were developed by summarising the evidence-based investigations. Evidence-based treatment options and their advantages and disadvantages were evaluated and rated to determine the strength of guideline recommendations (12).

The emergency department is of great significance in the treatment of paediatric fever, which is the most common reason for emergency department visits in children. The site, type and severity of infection can be quickly determined for the first time in the emergency department, allowing timely and effective treatment. Antibiotic treatment is initiated immediately after the definite diagnosis of the disease, which is essential in controlling the spread of the infection. Early use of appropriate antibiotics can effectively kill or inhibit the growth of pathogenic bacteria to prevent the development of more serious complications induced by infection (13). Paracetamol is the first choice in the treatment of patients with fever visiting the emergency department, followed by paracetamol combined with ibuprofen, which has a better efficacy in patients with bacterial fever (14). Comprehensive vital sign monitoring, laboratory tests and imaging examinations can be carried out in the emergency department to fully understand the condition of children, which helps doctors assess the health status of children more accurately and develop a scientific and rational treatment plan (15). C-reactive protein (CRP) can be used as a marker for immediate detection in children with fever. Procalcitonin (PCT) is produced rapidly when infection and tissue injury occur, and its specificity for bacterial infection is higher than other inflammatory markers, with a faster increase in abnormal values, making it the most accurate inflammatory marker currently available. Procalcitonin can be used for the early diagnosis of sepsis in children (16), and the combination of white blood cell count with CRP and PCT measurements can improve the diagnosis accuracy of bacterial infection in children (17). At the same time, emergency rescue and supportive treatment, including respiratory support, fluid resuscitation and electrolyte balance maintenance, are essential to maintain vital signs and organ functions in children during the acute phase. The significance of emergency department visits lies in the quick, comprehensive and scientific evaluation of the condition of children and the timely and effective treatments to maximise their survival and recovery rates. The emergency department is at the forefront of treatment and the key link in the protection of children's health.

In the Chinese literature, research priorities included clinical efficacy observation and efficacy evaluation of traditional or modern drugs in the treatment of paediatric fever; in the English literature, the priorities were the testing methods and vital sign evaluation in fevers induced by different causes. In some English-speaking countries, improving fever testing to determine the cause of diseases, including detailed temperature monitoring, laboratory tests and imaging examinations are emphasised in their healthcare system, and more precise treatment plans can be developed through comprehensive aetiological diagnoses. Articles in the English language reflected that this medical system emphasises the evaluation of drug treatment safety, particularly the potential side effects and interactions of drugs. Chinese-speaking doctors tend to quickly alleviate symptoms by medication and prefer to choose drugs based on the specific condition of the children to minimise the risk of adverse reactions, particularly when dealing with fever, which helps alleviate the discomfort symptoms of the child in the early stages of the disease. Traditional Chinese medicine is extremely significant in the treatment of paediatric fever. In a study by Huijuan Wang, “large pushing Tianheshui” manipulation was used to treat febrile rabbits, and the results showed that prolonged treatment with this technique accelerated the decrease in the rabbits' body temperature. Considering that the therapeutic effect of massage is positively correlated with the duration of manipulation within a certain time range, the antipyretic mechanism of this manipulation may involve the regulation of the balance of serotonin and norepinephrine in the hypothalamus (18). It has been demonstrated that the antipyretic six-method process can inhibit the activation of the COX-2/PGE2/EP3 signalling pathway in the hypothalamus caused by pyrogens, reduce the expression of cyclic adenosine monophosphate—a central positive regulatory mediator of body temperature (19)—and exert anti-inflammatory and antipyretic effects via inhibiting the TLR4/NF-κB pathway (20). Nursing plays a crucial role in the management of children with fever, and a suitable environment is crucial for the recovery of these patients. Nursing staff regularly adjust the room temperature, maintain ventilation and provide soft light and a quiet environment, which helps to alleviate the discomfort in children and give psychological support to ease their tension. Nursing staff also assist doctors in drug preparation and the implementation of physical therapies and cooperate closely with medical teams to ensure that comprehensive and meticulous nursing intervention is provided, which helps to improve the symptoms and recovery of children. Therefore, the quality requirements of nursing staff for health management are crucial for optimising the medical care quality and reducing the medical costs of children with fever (21).

The keyword burst analysis showed that bursts of the keywords “infantile massage”, “inflammatory factors” and “influenza A” were observed in 2020, and a burst of “paediatrics” started in 2021 and continued to 2023. Detection methods, such as blood culture and lumbar puncture, have also gradually been examined by researchers, and some scholars speculate that the fields involved in these keywords are worthy of attention in the future.

Research status and trends, as well as hotspots of paediatric fever, are reported in the present study. However, there were limitations in this study. The database processing method of CiteSpace software was relatively simple, though the VOSviewer tool was used in this study. In addition, the language and years of publications were defined, which limited the comprehensiveness of the analysed data. Future studies are needed to improve the analyses and studies in this field.

## 5 Conclusion

Based on the visual analysis results, several relatively stable research groups have been formed. Future studies on differential diagnosis, rational drug use, standardised management and clinical practice guidelines for paediatric fever are needed.

## Data Availability

The original contributions presented in the study are included in the article/Supplementary Material, further inquiries can be directed to the corresponding authors.
